# Timing within the oestrous cycle modulates adrenergic suppression of NK activity and resistance to metastasis: possible clinical implications

**DOI:** 10.1054/bjoc.2000.1563

**Published:** 2000-12

**Authors:** S Ben-Eliyahu, G Shakhar, K Shakhar, R Melamed

**Affiliations:** Department of Psychology, Tel Aviv University, Tel Aviv, 69978, Israel

**Keywords:** oestrous cycle, natural killer, catecholamines, metastasis, immunity

## Abstract

Clinical observations suggest that the rate of metastatic development and long-term mortality following surgery in breast cancer patients is influenced by the menstrual phase during which surgery is conducted. The menstrual cycle is known to modulate various physiological responses and medical conditions that involve adrenergic mechanisms (e.g., asthma). Natural killer activity (NKA), an immune function controlling metastasis, is suppressed following surgery, and in vitro by adrenaline. We therefore hypothesize that the clinical observation may be partly attributable to surgery-induced adrenergic suppression of NK-dependent resistance to metastasis, a suppression that depends on menstrual phase during surgery. To test this hypothesis in rats, 140 F344 females at different phases of their oestrous cycle were injected with a β-adrenergic agonist, metaproterenol (MP) (0.4 or 0.8 mg kg^–1^, s.c.), or with vehicle, before i.v. inoculation with MADB106 tumour cells. This syngeneic mammary adenocarcinoma line metastasizes only to the lungs, and is highly sensitive to NKA. In a second experiment, the suppression of NKA by MP was studied in vitro in blood drawn at different phases of the oestrous cycle (*n* = 36). Finally, the effects of stress on the number and activity of NK cells were assessed along the oestrous cycle (*n* = 71). The findings indicate that the suppressive effects of MP on resistance to metastasis and on NKA, are significantly greater during the oestrous phase characterized by high oestradiol levels (D3/proestrus/oestrus). Similarly, NKA per cell was suppressed by stress only during this phase. In untreated animals, in which inadvertent stress was minimized, no effects of the oestrous cycle on NKA or on resistance to metastasis were evident. These findings indicate that the oestrous cycle modulates adrenergic suppression of NKA and of resistance to metastasis. The relevance of these findings to the above clinical observation, as well as that of our related findings in women from a parallel study, is discussed. © 2000 Cancer Research Campaign http://www.bjcancer.com


*British Journal of Cancer* (2000) **83**(12), 1747–1754
© 2000 Cancer Research Campaign
doi: 10.1054/ bjoc.2000.1563, available online at http://www.idealibrary.com on IDEAL®

### Timing within the oestrous cycle modulates adrenergic suppression of NK activity and resistance to metastasis: possible clinical implications

**S Ben-Eliyahu, G Shakhar, K Shakhar and R Melamed**

Department of Psychology, Tel Aviv University, Tel Aviv 69978, Israel

**Summary** Clinical observations suggest that the rate of metastatic development and long-term mortality following surgery in breast cancer patients is influenced by the menstrual phase during which surgery is conducted. The menstrual cycle is known to modulate various physiological responses and medical conditions that involve adrenergic mechanisms (e.g., asthma). Natural killer activity (NKA), an immune function controlling metastasis, is suppressed following surgery, and in vitro by adrenaline. We therefore hypothesize that the clinical observation may be partly attributable to surgery-induced adrenergic suppression of NK-dependent resistance to metastasis, a suppression that depends on menstrual phase during surgery. To test this hypothesis in rats, 140 F344 females at different phases of their oestrous cycle were injected with a β-adrenergic agonist, metaproterenol (MP) (0.4 or 0.8 mg $kg^{-1}$, s.c.), or with vehicle, before i.v. inoculation with MADB106 tumour cells. This syngeneic mammary adenocarcinoma line metastasizes only to the lungs, and is highly sensitive to NKA. In a second experiment, the suppression of NKA by MP was studied in vitro in blood drawn at different phases of the oestrous cycle ($n$ = 36). Finally, the effects of stress on the number and activity of NK cells were assessed along the oestrous cycle ($n$ = 71). The findings indicate that the suppressive effects of MP on resistance to metastasis and on NKA, are significantly greater during the oestrous phase characterized by high oestradiol levels (D3/proestrus/oestrus). Similarly, NKA per cell was suppressed by stress only during this phase. In untreated animals, in which inadvertent stress was minimized, no effects of the oestrous cycle on NKA or on resistance to metastasis were evident. These findings indicate that the oestrous cycle modulates adrenergic suppression of NKA and of resistance to metastasis. The relevance of these findings to the above clinical observation, as well as that of our related findings in women from a parallel study, is discussed. © 2000 Cancer Research Campaign http://www.bjcancer.com

**Keywords:** oestrous cycle; natural killer; catecholamines; metastasis; immunity

An intriguing and controversial issue in surgical treatment of breast cancer is whether the timing of surgery in relation to the menstrual cycle affects the long-term rates of disease recurrence and survival. Several independent groups of researchers have reported an increase of up to 300% in the 10-year mortality rate of women undergoing surgery during days 2–14 of their menstrual cycle (Badwe et al, 1991; Senie et al, 1991; Saad et al, 1994). Other groups, however, have suggested that it is the perimenstrual period that is characterized by a higher mortality rate (Hrushesky et al, 1989), and several studies have failed to detect any relationship between the two variables (for review see Lemon and Rodriguez-Sierra, 1996). The considerable number of independent studies observing such a relationship suggests that this clinical phenomenon is authentic. However, this phenomenon may rely upon as yet unknown perioperative routines or circumstances, which invariably differ between hospitals.

Importantly, the increase in the rate of mortality was only found in women with positive lymph nodes, was due to malignant recurrence at remote locations, and occurred not before 3 years following surgery (Lemon and Rodriguez-Sierra, 1996). Thus, we suggest that the clinical phenomenon is related to metastatic development that is induced perioperatively and is modulated by the menstrual cycle during the perioperative period. Because the clinical phenomenon was not related to the expression of receptors for sex-steroid by the excised tumour (Lemon and Rodriguez-Sierra, 1996), it is not likely that it results from direct effects of sex hormones on the malignant tissue. Rather, it seems likely to result from alterations in host physiological mechanisms that affect metastatic development (e.g., levels of angiogenesis or immune competence).

Animal studies have indicated that natural killer cell activity (NKA) is an important immune function controlling the metastatic process (Brittenden et al, 1996), and clinical observations have supported these findings (Whiteside and Herberman, 1995). However, in order to suggest that alteration in NKA is a potential mechanism underlying the clinical phenomenon, it must be shown that the modulatory effects of the menstrual/oestrous cycle on NKA only become significant during the perioperative period. The menstrual cycle should have no significant effects on metastatic development outside the context of surgery. Otherwise, the effects of the specific menstrual phase on the day of surgery would be masked by the continuous effects of menstrual cycles preceding and following surgery.

A predominant effect of the menstrual phase during surgery may be a result of increased risk or increased susceptibility to

*Received 28 April 2000*
*Revised 16 October 2000*
*Accepted 17 October 2000*

*Correspondence to:* Dr Shamgar Ben-Eliyahu metastasis at this time. Several factors may contribute cumulatively to such increase in metastatic development. First, metastatic development could be promoted by a drop in levels of angiostatic agents due to the removal of the primary tumour, or initiated by the release of tumour emboli into circulation owing to the physical manipulation of the tumour during surgery. Second, immune suppression induced by surgical stress could render the organism susceptible to metastatic development that would otherwise have been kept under control. Indeed, marked suppression of NKA following surgical intervention is clinically well documented (Pollock et al, 1992; Beitsch et al, 1994; Brittenden et al, 1996; Shirakawa et al, 1998), and we have recently shown that such suppression in rats markedly promotes metastatic development (Ben-Eliyahu et al, 1999). Several studies have implicated sympathetic activation in suppressing NKA (Irwin et al, 1990; Brenner et al, 1992; Wu and Pruett, 1996; Ben-Eliyahu and Shakhar, 2000). For example, it was recently reported that the peripheral infusion of a β-adrenergic agonist suppressed NKA in rats, consequently causing a 30-fold increase in metastasis of a mammary adenocarcinoma (Shakhar and Ben-Eliyahu, 1998).

In patients, the perioperative period is characterized by marked sympathetic discharge. The activation of the sympathetic nervous system is induced by emotional and physical stress, certain anaesthetics, surgical hypothermia, pain and other factors. This characteristic of the perioperative period may contribute to the clinically established postoperative suppression of NKA. Indeed, catecholamines have been shown to suppress human NKA in vitro (Hellstrand and Hermodsson, 1989; Whalen and Bankhurst, 1990). Importantly, physiological responses and medical conditions that involve adrenergic mechanisms have been shown to be modulated by the menstrual cycle. For example, stress-induced increases in heart rate and blood pressure are more pronounced during the luteal phase of the menstrual cycle (Manhem et al, 1996; Litschauer et al, 1998), as are asthma attacks (Tan et al, 1997).

Therefore, in the current study we sought to assess whether adrenergic suppression of NKA and resistance to metastasis are also modulated by the oestrous cycle, and whether the oestrous cycle would fail to significantly affect NKA and resistance to metastasis in the absence of sympathetic activation. Only if both of these conditions are met, will it be possible to claim that the menstrual cycle modulates the intensity of NK suppression by catecholamines, and that this modulation may contribute to the clinically observed effects of the menstrual cycle on metastatic development following surgery. To this end, the effects of the oestrous cycle on NKA and resistance to metastasis were assessed both in the presence and in the absence of a β-adrenergic agonist. Three different approaches were used, each employing F344 female rats at different phases of their oestrous cycle: a) in vivo assessment of the metastatic efficacy of the MADB106 mammary adenocarcinoma line, following the administration of a β-adrenergic agonist, or in its absence. The MADB106 tumour is highly sensitive to NKA and metastasizes only to the lungs; b) ex-vivo assessment of the effects of swim stress on NKA measured in blood drawn following stress; and c) assessment of the in vitro impact of a β-adrenergic agonist on NKA in blood drawn at different phases of the oestrous cycle. Possible confounding effects of the inadvertent release of endogenous catecholamines were minimized by various measures.

### METHODS

### Animals

Fischer 344 (F344) female rats were purchased from Harlan Laboratories (Jerusalem, Israel), housed 4 per cage with free access to food and water, and kept under a 12:12 h light:dark cycle. At the time experiments were conducted, animals were 14–16 weeks old.

### Minimizing procedural stress and assessing vaginal cellularity

Prior to all experiments, rats were acclimatized to the vivarium for a minimum of 3 weeks. All experiments were conducted during the light phase, in which rats are inactive and their sympathetic tone is low. To reduce procedural stress, rats were habituated to the experimental routines. Specifically, all rats were handled for 6 consecutive days in a procedure room adjusted to the vivarium, and vaginal smears were taken starting from the fourth day. Following these 6 days of habituation, daily vaginal smears were obtained from all rats for 12 consecutive days during the first half of the light phase. On the twelfth day, at the same time as taking vaginal smears, the experimental procedures were conducted (i.e., metaproterenol/saline injection, blood withdrawal or tumour injection). Within a minute of the experimenter's entering the vivarium, the designated cage was moved into the procedure room, as per routine, and all 4 rats were injected subcutaneously, or simultaneously anaesthetized with halothane for tumour injection or blood withdrawal. Most commonly, all rats remained asleep when an experimenter entered the vivarium, and did not wake up before vaginal smears were taken or the experimental procedure initiated (on the twelfth day). When rats were returned to the vivarium following a procedure, they went back to sleep within less than 5 min. Following blood withdrawal, plasma was replaced with an artificial medium as soon as possible, in order to limit in vitro exposure of WBC to catecholamines that may have been released to the circulation in the minute proceeding blood withdrawal (e.g., during the 30 s of inducing the halothane anaesthesia).

### Determining the oestrous phase and duration of exposure to oestradiol

The 12 vaginal smears taken from each rat were stained (Wright stain, modified; Sigma Diagnostic WS 32) and analysed according to their cellular composition. The length of the last oestrous cycle in the experiment (either 4 or 5 days in the F344 rat), as well as the phase within the oestrous cycle on the day of the experiment, were determined according to the standard criteria described by Everett (Everett, 1989). As we have recently reported (Ben-Eliyahu et al, submitted), oestradiol levels start to increase during the light phase of the second day of diestrus, further increase during the third day of diestrus (which occurs only in 5-day cyclers), and reaches its highest levels during the light phase of the following proestrus day. On the following oestrus and diestrus-1 (metoestrus) days, levels of oestradiol are at their lowest. Thus, the following nomination system was designed in order to reflect duration of prior exposure to oestradiol, irrespective of the length of the oestrous cycle. A mark of +1E (one complete day of prior exposure to oestradiol – light phase to light phase) was given to 4-day cycler

 © 2000 Cancer Research Campaign rats on proestrus day, and to 5-day cyclers on the third day of dioestrus, as levels of oestradiol started to increase 24 h earlier in both cases. A mark of +2E was given to 4-day cyclers on oestrus day, and 5-day cyclers on proestrus day, as levels of oestradiol started to increase 48 h earlier in both cases. A mark of +3E was given to 5-day cyclers on oestrus day, as levels of oestradiol started to increase 72 h earlier. Although on the light phase of oestrus day, the levels of oestradiol are already low, a + sign (+2 or +3) is given because less than 24 h have elapsed since levels of oestradiol were high. Exposure to low levels of oestradiol were marked as 1no.E and 2no.E, indicating the lack of oestradiol exposure throughout the last day or the last 2 days, respectively. In both 4- and 5-day cyclers, dioestrus-1 (metoestrus) and dioestrus-2 were marked as 1no.E and 2no.E, respectively. In several figures and statistical analyses, the oestrous cycle was divided into high exposure to oestradiol versus low exposure to oestradiol, by collapsing the + days and the no.E days, respectively.

### Metaproterenol

Metaproterenol (MP) (Sigma, Israel): a non-selective β-adrenergic agonist, with a higher affinity to $\beta_2$ receptors than to $\beta_1$ and a half-life of about 2 h in rats (Muacevic, 1985). MP was dissolved in PBS for both s.c. injection and for in vitro use.

### Swim stress

A weight of 25 g $kg^{-1}$ of body weight was attached to the tails of stressed rats. Each stressed rat was then placed for 3 min in a tank containing water 35 cm deep at a temperature of 37°C, followed by a 3 min rest period. This procedure was repeated 5 times successively. All rats housed in a cage either served as controls or were stressed. Rats from the control groups were left undisturbed in the vivarium.

### Anti-NKR-P1 mAb

The anti-NKR-P1 (anti-rat NKR-P1A, Pharmingen, USA), originally termed mAb 3.2.3, binds to a surface antigen (NKR-P1) expressed on fresh and IL-2 activated NK cells in the rat, and, to a much lesser degree, on polymorphonuclear cells (Chambers et al, 1992). In vivo treatment of rats with anti-NKR-P1 selectively depletes large granular lymphocyte (LGL)/NK cells and eliminates NK and antibody dependent non-MHC-restricted cell cytotoxicity. T cell function and the percentage of T cells, monocytes, and PMNs are unaffected (Chambers et al, 1989; van den Brink et al, 1991). Conjugated with FITC, anti-NKR-P1 is used in FACS analysis to identify NK cells.

### Flow cytometry

An aliquot of 100 µl of blood was combined with 50 µl of PBS (supplemented with 2% FCS and 0.1% $NaN_3$) and 0.1 µg FITC-conjugated anti-NKR-P1. Samples were kept in the dark at room temperature thereafter. Following a 15 min incubation period, 2 ml FACs lysis solution was added (Becton Dickinson), and 10 min later, samples were centrifuged for 5 min at 500 ***g*** and the lysis solution was aspirated. Cells were washed again with 2 ml PBS (5 min centrifugation, 300 ***g***) and resuspended in 300 µl of PBS for flow cytometry analysis using a FACScan (Becton Dickinson).

The criterion for positive identification of spontaneously active LGL/NK cell was defined as being above a level of fluorescence intensity that distinguishes between bright and dim stained populations of NKR-P1 positive cells, as described previously by Chambers et al (Chambers et al, 1989, 1992). These previous studies also demonstrated that NKR-P1 is expressed by 94% of blood LGL cells and that the NK cytolytic activity was totally contained in the NKR-P1 bright cell population. Polymorphonuclear (PMN) leukocytes were found to express low levels of NKR-P1 and categorized as dim cells, and macrophages and mast cells were found to be negative (Chambers et al, 1989). In our studies, bright cells are defined as showing above 150 relative fluorescence intensity units, a level that distinguished between the two non-overlapping populations of the dim and bright NKR-P1$^+$ cells. Nonspecific binding was assessed using nonspecific IgG1 which consistently yielded 0% of brightly stained cells.

### Whole blood NKA assay

This 4 h cytotoxicity procedure assesses anti-tumour NKA per ml blood without prior purification of PBMCs (or the exclusion of any cell population). It reduces the time between blood withdrawal and assessment of cytotoxicity, and lessens the potential interference with NK cell function. Our previous studies (Page et al, 1994; Ben-Eliyahu et al, 1996) indicate that cytotoxicity in this assay depends on NK cells, since their selective depletion nullified all specific killing.

Blood was drawn into a heparinized syringe containing 25 units $ml^{-1}$ of blood. Exactly 1 ml of blood was washed once with PBS (diluted 1:4 volume/volume, centrifuged at 300 ***g*** for 10 min, and supernatant aspirated to original blood volume), and washed twice with complete media (RPMI 1640 media supplemented with 10% heat-inactivated FCS, 50 µg $ml^{-1}$ gentamicin, 2 mM L-glutamine, 0.1 mM non-essential amino acids, and 1 mM sodium pyruvate). For each of the 8 effector to target (E:T) ratios used, 100 µl of washed blood was placed into each well of a microtitre plate and 150 µl of $^{51}$Cr-labelled YAC-1 tumour cells in complete media was added on top of the blood. A concentration of $3.2 \times 10^6$ $ml^{-1}$ YAC-1 was used for the lowest E:T ratio (approximately 1:16 NK:YAC-1, depending on individual number of NK cells $ml^{-1}$ of blood) and sequentially diluted by two to produce higher E:T ratios (approximately 8:1 at the highest). Spontaneous (SP) and maximal (MAX) releases of $^{51}$Cr from target cells were determined by substituting blood with complete media or TRITON X-100 (Sigma, Israel), respectively. Plates were centrifuged at 500 ***g*** for 10 min to create a buffy coat layer of leukocytes and target cells on top of the red blood cells prior to a 4 h incubation period. Following incubation, plates were again centrifuged and aliquots of 100 µl of the supernatant were recovered from each well for assessment of radioactivity in a γ-counter. SP and MAX release of radioactivity from tumour cells were measured separately for each of the 6 tumour concentrations, and percent specific lysis was calculated for each E:T ratio using the formula $(0.8 \times X-SP)/MAX-SP) \times 100$, where X is the experimental release. The X value is multiplied by 0.8 to correct for the reduction in the supernatant volume into which $^{51}$Cr is released. This reduction is caused by the presence of RBCs in the wells (MAX and SP are assessed in the absence of RBCs). The exact E:T ratio were

© 2000 Cancer Research Campaign 

individually calculated for each rat according to its number of NK cells determined by the FACS analysis. The level of cytotoxicity for each of the 8 NK:YAC-1 ratio used for statistics and for graph presentation were calculated using a regression exponential fit method (Pollock et al, 1990).

### Radiolabelling of YAC-1 target cells

40 × $10^6$ cells YAC-1 cells were incubated for 1 h with 200 μCi $^{51}$Cr (in 200 μl saline), 400 μl FCS, and 300 μl complete media. Following incubation, cells were washed 3 times (300 ***g***, for 10 min) and adjusted to the desired concentration in complete media.

### MADB106 tumour line

MADB106 is a selected variant cell line obtained from a pulmonary metastasis of a mammary adenocarcinoma (MADB100) chemically induced in the inbred F344 rat (Barlozzari et al, 1985). Following i.v. inoculation, MADB106 tumour cells seed and colonize only to the lungs, and the number of tumour cells retained in the lung 24 h following inoculation, as well as the consequent metastases enumerated weeks later are highly dependent on NK activity (Barlozzari et al, 1985; Ben-Eliyahu and Page, 1992).

The MADB106 cell line was maintained in monolayer cultures in complete media and separated from the flask using 0.25% trypsin.

### Radiolabelling of MADB106 tumour cells and assessment of lung tumour retention

For assessment of lung tumour retention, DNA radiolabelling of tumour cells was accomplished by adding 0.4 mCi $ml^{-1}$ of $^{125}$Iododeoxyuridine ($^{125}$IDUR) (ICN Radiochemicals, Irvine, CA, USA) to the growing cell culture one day before harvesting the cells for injection.

For tumour cell injection, rats were lightly anaesthetized with halothane, and 4 × $10^5$ $kg^{-1}$ $^{125}$IDUR-labelled MADB106 tumour cells in approximately 0.5 ml of PBS were injected into their tail vein. 9 h later, rats were euthanized with halothane, and their lungs removed and placed in a gamma-counter for assessment of radioactive content. The percentage of tumour cells retained was calculated as the ratio of radioactivity measured in the lungs to total radioactivity in the injected tumour cells suspension. Our previous studies have indicated that the levels of lung radioactivity reflect the numbers of viable tumour cells in the lungs (for more information see Ben-Eliyahu & Page, 1992).

### Group assignment, counterbalancing and statistics

Rats did not synchronize their oestrous cycle within or between cages in any of the experiments, as was the case in our previous studies (Ben-Eliyahu et al, 1996). About half of the rats had a cycle of 4 days and half of 5 days. Approximately even numbers of rats were in each of the oestrous phases/days during the day of the experiment, and the experimenters were naive to the rats' oestrous phase. The time and order of MP/saline injection, blood withdrawal, and tumour injection were counterbalanced across all groups in all experiments (i.e., conducted in parallel in all groups). For each experiment, the relevant procedures were completed in all animals within less than 90 minutes. For statistical analysis, ANOVA was conducted, and, provided significant group differences existed, Scheffe or Bonferroni post hoc tests were used to identify specific differences. α was set to 0.05 in all experiments.

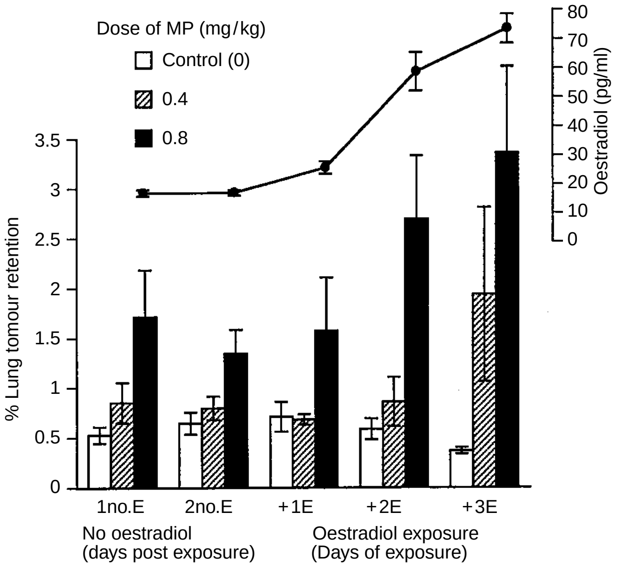

**Figure 1** The effects of the β-adrenergic agonist MP, and of the number of days of pre-exposure to oestradiol along the oestrous cycle, on lung tumour retention of the MADB106. Number of days of exposure to high levels of oestradiol prior to MADB106 inoculation is noted +1E, +2E and +3E, and the number of complete days following the decline of oestradiol is noted 1no. E and 2no.E (see Methods for details). The line represents levels of oestradiol in F344 rats one day before the administration of the MADB106 (adopted from Ben-Eliyahu et al, submitted). Both oestradiol levels and lung tumour retention reflect the average across the light phase of each day

### PROCEDURES AND RESULTS

### Exp. 1: The effects of MP and the oestrous cycle on MADB106 lung tumour retention (LTR)

On the day of the experiment, 140 rats at different oestrous phases were injected s.c. with either saline (control), 0.4 mg $kg^{-1}$ MP, or 0.8 mg $kg^{-1}$ MP. One h later, radiolabelled MADB106 cells were injected i.v. under light halothane anoesthesia. 9 h later rats were euthanized with halothane and lungs removed for the assessment of LTR. The experiment was conducted in two replicates, each using 70 rats representing all experimental groups.

### Results

The two replicates yielded a very similar pattern of effects, and thus were combined. To overcome daily differences in baseline levels of LTR between the 2 replicates, results from the second replicate were multiplied by a factor of 2.3, equalizing the average levels of saline-treated animals in the 2 replicates. Rats were approximately evenly distributed across the different days of the oestrous cycle, and categorized as 'High' or 'Low' for exposure to oestradiol as described above.

Whereas no significant effects of the oestrous cycle on baseline levels of LTR were evident, marked effects of the oestrous cycle were revealed in rats injected with MP. The LTR-increasing effects of MP were proportional to the duration of exposure to oestradiol (Figure 1), and were significantly larger during the high oestradiol

 *© 2000 Cancer Research Campaign*

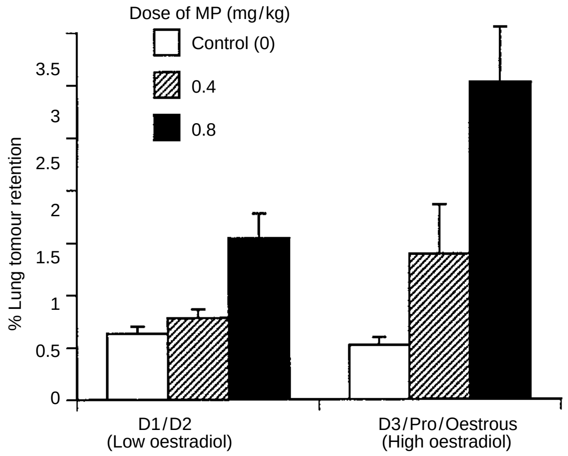

**Figure 2** The effects of the β-adrenergic agonist MP, and of the oestrous cycle, on lung tumour retention of the MADB106. The oestrous cycle was divided into days of prior exposure to high levels of oestradiol, diestrus-3, proestrus and oestrus (D3, pro, oestrus), versus days of prior low levels of oestradiol, dioestrus-1 and dioestrus-2 (D1 and D2). The effects of MP were markedly larger during the high oestradiol period compared to the low oestradiol period, as indicated by a significant interaction between the effects of MP and of the oestrous cycle. No effect of the oestrous cycle was evident in the absence of MP (Control)

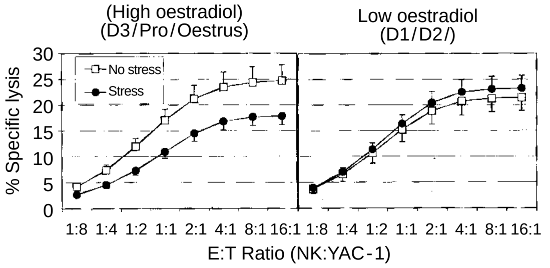

**Figure 3** The effects of the oestrous cycle and of swim stress on NKA. The oestrous cycle was divided into days of prior exposure to high levels of oestradiol, diestrus-3, proestrus and oestrus (D3, pro, oestrus), versus days of prior low levels of oestradiol, dioestrus-1 and dioestrus-2 (D1 and D2). Swim stress suppressed NKA during the high oestradiol period, but not during the low oestradiol period, as indicated by a significant interaction between the effects of stress and of the oestrous cycle. No significant effect of the oestrous cycle was evident within control (No stress) rats

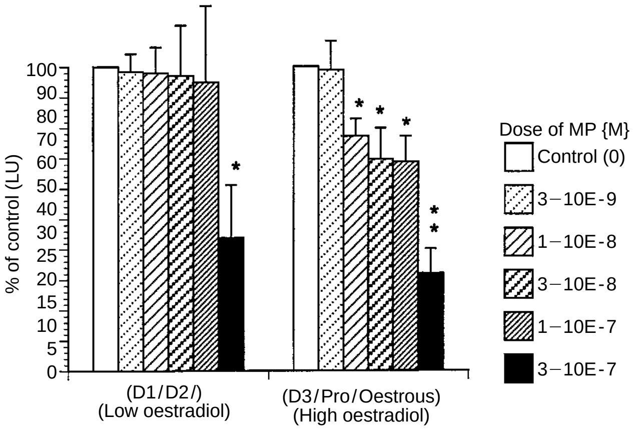

**Figure 4** The effects of the oestrous cycle and in vitro exposure to the β-adrenergic agonist, MP, on NKA. NKA is presented as % of control (LUMP/LUcontrol). The oestrous cycle was divided into days of prior exposure to high levels of oestradiol, diestrus-3, proestrus and oestrus (D3, pro, oestrus), versus days of prior low levels of oestradiol, diestrus-1 and diestrus-2 (D1 and D2). The in vitro effects of MP were markedly larger if blood was drawn during the high oestradiol period compared to the low oestradiol. Significant suppression of NKA was evident in the 4 higher doses of MP in the high oestradiol condition, but only in the highest dose of MP in the low oestradiol condition. No effect of the oestrous cycle was evident in the absence of MP (not shown in the figure)

exposure period compared to the low oestradiol exposure period (Figure 2). Specifically, two-way ANOVA revealed a significant main effect for MP ($F_{(2,134)} = 16.7$, $P < 0.05$), a significant main effect for exposure to oestradiol (high vs. low) ($F_{(1,134)} = 5.2$, $P < 0.05$), as well as a significant interaction between these 2 factors ($F_{(2,134)}$) = 3.4, $P < 0.05$). Post hoc Scheffe comparison indicated that in rats injected with 0.8 mg $kg^{-1}$ MP, LTR was significantly higher during the high oestradiol period then during the low oestradiol period ($P = 0.01$).

### Exp. 2: The effects of swim stress and the oestrous cycle on the number and activity of NK cells

On the day of the experiment, 44 rats were exposed to swim stress, and 27 rats remained undisturbed in their home cages. Two hours following stress, 1.5–2 ml blood was simultaneously drawn from the stressed and control rats. Blood was drawn by cardiac puncture under light halothane anaesthesia using a 25 G needle. The number of NK cells per ml of blood and activity per NK cells were assessed thereafter in all 71 rats, as described above.

### Results

Of the 71 rats, 41 were categorized as 'high' exposure to oestradiol (26 were stressed and 15 were control), and 30 were categorized as 'low' exposure to oestradiol (18 were stressed and 12 were control). Repeated measure ANOVA (for the 8 E:T ratio) indicated that swim stress significantly suppressed NK activity per NK cells in the high oestradiol group ($F(1,273) = 6.4$, $P < 0.05$), whereas no effect of swim stress was evident in the low oestradiol group ($F(1,196) = 0.24$, $P = 0.65$) (Figure 3). A 2 × 2 repeated measure ANOVA indicated a significant interaction between the effects of stress and exposure to oestradiol ($F(1,469) = 3.93$, $P < 0.05$). No significant oestrous cycle effects on baseline levels of NKA were evident. The numbers of NK cells were not significantly affected by swim stress or by the oestrous cycle, nor was there an interactive effect between these two factors.

### Exp. 3: The in vitro effects of MP and of the oestrous cycle on NKA

On the day of the experiment, 5–8 ml blood was drawn by cardiac puncture under halothane anaesthesia from 35 rats at different oestrous phases, and serum was replaced with complete media as described in the procedure for assessing NKA. Different doses of MP (to achieve final concentrations of $1 \times 10^{-7}$, $3 \times 10^{-7}$, $1 \times 10^{-8}$, $3 \times 10^{-8}$, or $1 \times 10^{-9}$ M in the NK assay) were added to different aliquots of 1 ml blood from each rat. Two additional aliquots from each rat were used as control and were supplemented with vehicle. One h later, NKA was assessed as described above, without additional 'washing' of the blood. The experiment was conducted in two replicates, using 12 and 24 rats.

© 2000 Cancer Research Campaign

### Results

Of the 36 rats, 22 were categorized as 'high' and 14 as 'low' for exposure to oestradiol. To compare NKA between the different doses of MP, lytic units (LU) were calculated for each rat using the formula 100/$ET_{1/3}$, where $ET_{1/3}$ is the E:T ratio needed to reach a 1/3 increment in target cytotoxicity (along the different E:T ratios) in the aliquots of blood not exposed to MP (average of both replicates). The regression exponential fit method (Pollock et al, 1990) was used to infer $ET_{1/3}$ from the data. Data were converted to a percentage of control levels in each rat, maintaining the 2 replicates of 0 levels of MP as 2 different numbers to indicate assay errors. Whereas in the high group, MP significantly suppressed NKA in a dose-dependent manner beginning from the second dose, in the Low group only the highest dose of MP had a significant effect (Figure 4). ANOVA indicated significant main effects of MP dose ($F(5,195) = 13.3$, $P < 0.05$) and of exposure to oestradiol ($F(1,195) = 4.9$, $P < 0.05$), and Bonferroni post hoc comparison ($\alpha$ corrected to 0.00511 for the 10 comparisons) indicated significantly lower NKA in the 4 higher doses in the High estradiol condition, but only in the highest dose of MP in the Low estradiol condition. Baseline levels of NKA (no MP) along the oestrous cycle did not show significant differences.

### DISCUSSION

As in our previous studies, β-adrenoceptor stimulation and stress suppressed NKA and host resistance to metastasis. The main finding of the current study is that this suppression was markedly modulated by the oestrous cycle. Specifically, on days diestrus-3, proestrus, and oestrus, the metastasis-enhancing effects of the β-adrenergic agonist, metaproterenol (MP), were significantly greater than on days diestrus-1 (metoestrus) and diestrus-2. Similarly, swim stress suppressed NKA only during this 3-day period, and markedly lower concentrations of MP were sufficient to suppress NKA in vitro if blood was drawn during these 3 days. On the other hand, when rats were not stressed or not challenged with MP, the oestrous cycle had no effect on NKA or levels of resistance to metastasis.

Because we hypothesized that the effects of the oestrous cycle would be restricted to conditions of adrenoceptor stimulation, we made a deliberate effort to minimize procedural stress in the control groups. Additionally, in order to avoid unplanned exposure of leukocytes to catecholamines secreted upon blood withdrawal, plasma was rapidly replaced by an artificial medium (see Methods). Such measures were not taken in previous studies, in which we and others found oestrous cycle effects on NKA and resistance to metastasis without deliberately exposing rats to stress hormones (White et al, 1982, 1997; Sulke et al, 1985; Ratajczak et al, 1988; Ben-Eliyahu et al, 1996; Page and Ben-Eliyahu, 1997). Based on the findings of the current study, in which the effects of the oestrous cycle are shown to be limited to conditions of stress or adrenoceptor stimulation, we suggest that the previous findings are attributable to unintended procedural stress and the systemic release of catecholamines.

The in vitro study, in which MP was added to blood drawn at different oestrous phases, directly and clearly indicates that the suppression of NKA by β-adrenergic stimulation is modulated by the oestrous cycle. We have conducted the same study in women and found homologous findings. Specifically, 3-fold higher concentrations of MP were needed to achieve the same levels of NK suppression in blood drawn during the follicular phase compared to blood drawn during the luteal phase (Shakhar et al, 2000). In other studies, the luteal phase has been reported to be characterized by a higher lymphocyte expression of $\beta_2$-adrenoceptors, and by a higher cAMP response to isoprenaline (Wheeldon et al, 1994). Suppression of NKA by adrenergic agonists, as well as by other immunomodulators (e.g. prostaglandin-E2), is known to be mediated by elevated cAMP (Whalen and Bankhurst, 1990). Therefore, we suggest that alterations in the expression of β-adrenoceptors and their functional response, underlie the modulatory effects of the ovulatory cycle on the suppression of NKA by β-adrenergic agents in human and murine NK cells.

The in vivo study indicated that the oestrus cycle modulates resistance to metastasis under the condition of β-adrenoceptor activation. We propose that this effect is mediated by oestrous modulation of the suppressive effects of MP on NK activity. Firstly, the MADB106 line is highly sensitive to NKA in vivo. Specifically, selective depletion of NK cells causes an approximately 200-fold increase in MADB106 lung tumour retention and the number of lung metastases (Ben-Eliyahu et al, 1991; Ben-Eliyahu and Page, 1992; Shakhar and Ben-Eliyahu, 1998). Secondly, we have recently reported various findings indicating that the increase in MADB106 metastasis caused by the administration of MP, with the same timing and dosage used here, is mediated by suppression of NKA. For example, selective depletion of NK cells prevented the metastasis-enhancing effects of MP (without causing a ceiling effect) (Shakhar and Ben-Eliyahu, 1998). Therefore, the modulatory effects of the oestrous cycle on the metastasis-enhancing effects of MP, are likely to be mediated by oestrus modulation of adrenergic suppression of NKA. Such modulation of NKA was indeed demonstrated in vitro in the current study in rats, and in our corresponding study in women (Shakhar et al, 2000).

The swim stress paradigm was chosen because it reliably suppresses NKA per NK cell and consequently increases metastasis of the MADB106 line (Ben-Eliyahu et al, 1991, 1999, in press). This paradigm is known to induce the release of both glucocorticoids and catecholamines. However, the metastasis-enhancing effects of this paradigm, as well as several other stress paradigms, are mediated by catecholamines rather than glucocorticoids (Ben-Eliyahu et al, in press). We have also shown that administration of adrenaline or MP in rats suppresses NKA, and that the NK suppressive effects of this stress paradigm are mediated by the release of adrenal catecholamines (Shakhar and Ben-Eliyahu, 1998; Ben-Eliyahu et al, in press). Therefore, we suggest that the current NK suppressive effects of swim stress, which are modulated by the oestrous cycle in female F344 rats, are also mediated by the systemic release of catecholamines. In this experiment, we used a stress paradigm, rather than a systemic injection of MP, in order to make our finding pertinent to the more complex stress-induced changes in the physiological milieu. In order to further simulate the clinical situation and to assess the role of adrenergic and other mechanisms in mediating the effects of surgery, our ongoing studies employ spontaneously metastasizing tumours and their surgical excision.

Several lines of evidence suggest that oestradiol is a prominent hormonal mediator of the current effects of the oestrous cycle, and that its impacts are manifested approximately one day following its increase, and terminate one day after its decline (a delay which also characterizes other effects of oestradiol). Firstly, as seen in Figure 1, the susceptibility to metastasis parallels serum

 © 2000 Cancer Research Campaign levels of oestradiol on the day before tumour inoculation. We have recently conducted a comprehensive study of the levels of sex hormones along the oestrous cycle in the F344 rat, and found that no other sex hormone is synchronized with the susceptibility to metastasis as closely as oestradiol (Ben-Eliyahu et al, submitted). Specifically, progesterone has two distinct peaks of similar size along the oestrous cycle, and LH, FSH, prolactin, testosterone, and corticosterone levels begin their increase on proestrus day, after susceptibility to metastasis has already increased. Secondly, in a previous study, we have shown that the infusion of physiological levels of oestradiol, but not progesterone, to ovariectomized F344 females, increased susceptibility to MADB106 metastasis. This effect was evident only 24 h after the infusion of oestradiol (Ben-Eliyahu et al, 1996), as was also the case in the current study in which the effects were evident only 24 h after the rise in oestradiol levels. Thirdly, expression of β-adrenergic receptors on women's lymphocytes was found to be higher during days of elevated oestradiol levels (days 21–23 vs. days 2–4) (Wheeldon et al, 1994), although in humans this period is also characterized by high levels of progesterone. Lastly, our corresponding findings in women indicate that the in vitro suppressive effects of MP on NKA were better correlated with levels of oestradiol than with levels of progesterone (Shakhar et al, 2000). Nevertheless, direct evidence is required to confirm the role of oestradiol and to assess the potential contribution of other hormones.

It is interesting to assess the relevance of our findings to the clinical phenomenon. In clinical settings, specific periods of the menstrual cycle during which breast cancer was surgically removed, have been associated with a higher risk of metastasis and mortality (Lemon and Rodriguez-Sierra, 1996). Our studies in rats are limited to a single (although important) immune function controlling metastasis, one mammary tumour line, and the response to adrenergic challenge. Unfortunately, the timing of the increased impact of adrenergic agents indicated by our animal and human studies (during and shortly after exposure to oestradiol), cannot be easily compared to the timing of the high-risk period observed clinically, as different clinical reports suggest different menstrual phases as being the high-risk period (Lemon and Rodriguez-Sierra, 1996). However, the apparent inconsistency within the clinical literature, and the lack of an exact time concurrence between our findings and the clinical observations, do not create a major difficulty in relating our findings to the clinical phenomenon, as both apparent inconsistencies may be the result of hospital variation in perioperative routines. For example, it may be suggested that the biopsy itself and the stress it induces promotes the dissemination of the tumour, more so when radical biopsy is conducted. Different hospitals may employ different methods of biopsy and conduct them at a uniform but different time intervals before surgery. Therefore, given our finding in rats and humans, we propose the following mechanism as underlying, at least partly, the clinical observation. Surgery itself (or a radical biopsy) promotes the metastatic process, while certain aspects of the menstrual cycle (e.g., levels of specific sex hormones) modulate the patient's resistance to metastasis in these vulnerable conditions. Specifically, we suggest that a mechanism contributing to the promotion of metastasis by surgery is an adrenergic suppression of NK activity. Indeed, suppression of NKA is well documented clinically following surgery (Pollock et al, 1992; Beitsch et al, 1994; Shirakawa et al, 1998), has been suggested by animal and human studies to promote metastasis (Brittenden et al, 1996; Ben-Eliyahu et al, 1999), and is shown here and in our study in women to be modulated by the oestrous and menstrual cycles.

A possible implication of the present findings and our interpretations, is that the prevention of adrenergically-mediated immune suppression would reduce the need to control the timing of surgery in premenopausal women. Since adrenergic suppression of immune competence is probably not restricted to the surgical effects of operating on breast cancer patients, limiting the immune suppression induced by surgery may benefit all patients undergoing intrusive procedures while bearing metastasizing tumours.

### REFERENCES

Badwe RA, Gregory WM, Chaudary MA, Richards MA, Bentley AE, Rubens RD and Fentiman IS (1991) Timing of surgery during menstrual cycle and survival of premenopausal women with operable breast cancer. *Lancet* **337**: 1261–1264

Barlozzari T, Leonhardt J, Wiltrout RH, Herberman RB and Reynolds CW (1985) Direct evidence for the role of LGL in the inhibition of experimental tumor metastases. *Journal of Immunology* **134**: 2783–2789

Beitsch P, Lotzova E, Hortobagyi G and Pollock R (1994) Natural immunity in breast cancer patients during neoadjuvant chemotherapy and after surgery. *Surgical Oncology* **3**: 211–219

Ben-Eliyahu S and Page GG (1992) In vivo assessment of natural killer cell activity in rats. *Progress in NeuroEndocrine Immunology* **5**: 199–214.

Ben-Eliyahu S and Shakhar G (2000) The impact of stress, catecholamines, and the menstrual cycle on NK activity and tumor development: from in vitro studies to biological significance. In: *Psychoneuroimmunology* Ader R, Felten DL and Cohen N (eds), Vol. 2. pp. 545–563. Academic Press: San Diego

Ben-Eliyahu S, Yirmiya R, Liebeskind JC, Taylor AN and Gale RP (1991) Stress increases metastatic spread of a mammary tumor in rats: evidence for mediation by the immune system. *Brain, Behavior, and Immunity* **5**: 193–205

Ben-Eliyahu S, Page GG, Shakhar G and Taylor AN (1996) Increased susceptibility to metastasis during pro-oestrus/oestrus in rats: possible role of oestradiol and natural killer cells. *British Journal of Cancer* **74**: 1900–1907

Ben-Eliyahu S, Page GG, Yirmiya R and Shakhar G (1999) Evidence that stress and surgical interventions promote tumor development by suppressing natural killer cell activity. *Int J Cancer* **80**: 880–888

Ben-Eliyahu S, Haim S, Shakhar G and Rossene E (submitted) Levels of sex hormones in 4 and 5-days cycling Fischer 344 rats and their simulation in ovariectomized rats.

Ben-Eliyahu S, Shakhar G, Page GG, Stefanski V and David K (in press). Suppression of NK activity and resistance to metastasis by stress: A role for adrenal catecholamines and β-adrenoceptors *NeuroImmunoModulation*

Brenner GJ, Felten SY, Felten DL and Moynihan JA (1992) Sympathetic nervous system modulation of tumor metastases and host defense mechanisms. *Journal of Neuroimmunology* **37**: 191–201

Brittenden J, Heys SD, Ross J and Eremin O (1996) Natural killer cells and cancer. *Cancer* **77**: 1226–1243

Chambers WH, Vujanovic NL, DeLeo AB, Olszowy MW, Herberman RB and Hiserodt JC (1989) Monoclonal antibody to a triggering structure expressed on rat natural killer cells and adherent lymphokine-activated killer cells. *Journal of Experimental Medicine* **169**: 1373–1389

Chambers WH, Brumfield AM, Hanley-Yanez K, Lakomy R, Herberman RB, McCaslin DC, Olszowy MW and McCoy JP, Jr (1992) Functional heterogeneity between NKR-P1$^{bright}$/Lycopersicon esculentum lectin (L.E.)$^{bright}$ and NKR-P1$^{bright}$/L.E.$^{dim}$ subpopulations of rat natural killer cells. *Journal of Immunology* **148**: 3658–3665

Everett JW (1989) Neurobiology of reproduction in the female rat. A fifty-year perspective. *Monographs on Endocrinology* **32**: 1–133

Hellstrand K and Hermodsson S (1989) An immunopharmacological analysis of adrenaline-induced suppression of human natural killer cell cytotoxicity. *International Archives of Allergy and Applied Immunology* **89**: 334–341

Hrushesky WJ, Bluming AZ, Gruber SA and Southern RB (1989) Menstrual influence on surgical cure of breast cancer. *Lancet* **2**: 949–952

Irwin M, Hauger RL, Jones L, Provencio M and Britton KT (1990) Sympathetic nervous system mediates central corticotropin-releasing factor induced suppression of natural killer cytotoxicity *Journal of Pharmacology and Experimental Therapeutics* **255**: 101–107

Lemon HM and Rodriguez-Sierra JF (1996) Timing of breast cancer surgery during the luteal menstrual phase may improve prognosis. *Nebr Med J* **81**: 110–115

© 2000 Cancer Research Campaign

Litschauer B, Zauchner S, Huemer KH and Kafka-Lutzow A (1998) Cardiovascular, endocrine, and receptor measures as related to sex and menstrual cycle phase. *Psychosom Med* **60**: 219–126

Manhem K, Hansson L, Milsom I, Pilhall M and Jern S (1996) Estrogen and progestagen modify the hemodynamic response to mental stress in young women *Acta Obstet Gynecol Scand* **75**: 57–62

Muacevic G (1985) Determination of bioavailability on the basis of tachycardia after intravenous and oral administration of fenoterol, orciprenaline and salbutamol in non-anaesthetized rats. *Arzneimittelforschung* **35**: 406–408

Page GG and Ben-Eliyahu S (1997) Increased surgery-induced metastasis and suppressed natural killer cell activity during proestrus/estrus in rats. *Breast Cancer Res Treat* **45**: 159–167

Page GG, Ben-Eliyahu S and Liebeskind JC (1994) The role of LGL/NK cells in surgery-induced promotion of metastasis and its attenuation by morphine. *Brain, Behavior, and Immunity* **8**: 241–250

Pollock RE, Zimmerman SO, Fuchshuber P and Lotzova E (1990) Lytic units reconsidered: pitfalls in calculation and usage. *Journal of clinical laboratory anlaysis* **4**: 274–282

Pollock RE, Lotzova E and Stanford SD (1992) Surgical stress impairs natural killer cell programming of tumor for lysis in patients with sarcomas and other solid tumors. *Cancer* **70**: 2192–202

Ratajczak HV, Sothern RB and Hrushesky WJ (1988) Estrous influence on surgical cure of a mouse breast cancer. *Journal of Experimental Medicine* **168**: 73–83

Saad Z, Bramwell V, Duff J, Girotti M, Jory T, Heathcote G, Turnbull I, Garcia B and Stitt L (1994) Timing of surgery in relation to the menstrual cycle in premenopausal women with operable breast cancer. *British Journal of Neurosurgery* **81**: 217–220

Senie RT, Rosen PP, Rhodes P and Lesser ML (1991) Timing of breast cancer excision during the menstrual cycle influences duration of disease-free survival. *Annales De Medecine Interne* **115**: 337–342

Shakhar G and Ben-Eliyahu S (1998) In vivo beta-adrenergic stimulation suppresses natural killer activity and compromises resistance to tumor metastasis in rats. *Journal of Immunology* **160**: 3251–3258

Shakhar K, Shakhar G, Rosenne E and Ben-Eliyahu S (2000). Timing within the menstrual cycle, sex, and the use of oral contraceptives determine adrenergic suppression of NK cell activity. *British Journal of Cancer* **83**: 1630–1636

Shirakawa T, Tokunaga A and Onda M (1998) Release of immunosuppressive substances after gastric resection is more prolonged than after mastectomy in humans. *Int Surg* **83**: 210–214

Sulke AN, Jones DB and Wood PJ (1985) Hormonal modulation of human natural killer cell activity in vitro. *J Reprod Immunol* **7**: 105–110

Tan KS, McFarlane LC and Lipworth BJ (1997) Loss of normal cyclical beta 2 adrenoceptor regulation and increased premenstrual responsiveness to adenosine monophosphate in stable female asthmatic patients. *Thorax* **52**: 608–611

van den Brink MR, Hunt LE and Hiserodt JC (1991) In vivo treatment with monoclonal antibody 3.2.3 selectively eliminates natural killer cells in rats. *Journal of Experimental Medicine* **171**: 197–210

Whalen MM and Bankhurst AD (1990) Effects of beta-adrenergic receptor activation, cholera toxin and forskolin on human natural killer cell function. *Biochemical Journal* **272**: 327–331

Wheeldon NM, Newnham DM, Coutie WJ, Peters JA, McDevitt DG and Lipworth, BJ (1994) Influence of sex-steroid hormones on the regulation of lymphocyte beta 2-adrenoceptors during the menstrual cycle. *British Journal of Clinical Pharmacology* **37**: 583–588

White D, Jones D, Cooke T and Kirkham N (1982) Natural killer (NK) activity in peripheral blood lymphocytes of patients with benign and malignant breast disease. *British Journal of Cancer* **46**: 611–616

White HD, Crassi KM, Givan AL, Stern JE, Gonzalez JL, Memoli VA, Green WR and Wira CR (1997) CD3+ CD8+ CTL activity within the human female reproductive tract: influence of stage of the menstrual cycle and menopause. *J Immunol* **158**: 3017–3027

Whiteside TL and Herberman RB (1995) The role of natural killer cells in immune surveillance of cancer. *Curr Opin Immunol* **7**: 704–710

Wu WJ and Pruett SB (1996) Suppression of splenic natural killer cell activity in a mouse model for binge drinking. II. Role of the neuroendocrine system. *Journal of Pharmacology and Experimental Therapeutics* **278**: 1331–1339

*© 2000 Cancer Research Campaign*
